# The effect of preoperative embolization rate on surgical outcomes for carotid paraganglioma resection

**DOI:** 10.1590/1806-9282.20240371

**Published:** 2024-08-16

**Authors:** Mustafa Yildirim, Hanefi Yildirim, Yusuf Doğan

**Affiliations:** 1 Fırat University, Faculty of Medicine, Department of Radiology – Elâzığ, Turkey.

**Keywords:** Carotid body tumor, Vascular complication, Artery embolization

## Abstract

**OBJECTIVE:**

Preoperative embolization of paragangliomas decreases tumor volume and reduces intraoperative blood loss. This study aimed to evaluate the effect of the rate of devascularization achieved by preoperative embolization of carotid body tumors on surgical outcomes.

**METHODS:**

Patients with carotid body tumors who underwent preoperative transarterial embolization between 2013 and 2024 were included in this retrospective study. The Shamblin classification of all patients was carried out using radiological imaging. Devascularization rates obtained after the embolization of carotid body tumors were determined from angiographic images. Patients were divided into two groups: near-complete embolization (devascularization rate >90%) and incomplete embolization (devascularization rate <90%). Hemoglobin loss was calculated with blood tests before and immediately after surgery. Tumor volume loss was calculated by preoperative radiological tumor volume and postoperative surgical specimen volume. Hemoglobin loss, tumor volume loss, and postoperative complication rates of the two groups were compared.

**RESULTS:**

A total of 31 patients with carotid body tumors who underwent surgery were included in the study. Near-complete embolization was achieved in 21 patients (67.74%), while incomplete embolization was achieved in 10 patients (32.25%). Shamblin classification was statistically similar (p>0.05) between the two groups. The vascular complication rate in the near-complete embolization group was significantly lower than in the incomplete embolization group (p=0.027). However, no significant difference was observed in neurological complication rates, hemoglobin loss, and tumor volume loss parameters between the two groups (p>0.05).

**CONCLUSION:**

The preoperative devascularization rate should be at least 90% to minimize the risk of vascular complications.

## INTRODUCTION

Carotid body tumors are the most common head-neck paraganglioma tumors. They are highly vascular, rare, and generally benign tumors^1,2^. The predicted incidence of carotid body tumors is 1:30,000 and accounts for 3% of paragangliomas^3^. They often present as a painless, slow-growing lateral neck lump.

Treatment options include conservative management, resection, and radiotherapy. The only curative treatment for these tumors is surgical resection. Multiple difficulties arise in the surgical treatment of carotid body tumors, which are mostly due to their complex anatomical location and high vascularity. The Shamblin classification is used to evaluate the extent of difficulty in the surgical resection of the tumor. Involvement of the internal carotid artery (ICA) and external carotid artery (ECA) in the tumor can also be evaluated with the intraoperative Shamblin classification. Preoperative prediction of the Shamblin classification can be achieved by assessing the angle of ICA to tumor contact in radiological imaging (group I: <180°, group II: <180–270°, and group III: >270°)^4^.

Preoperative carotid body tumor embolization is a standard step in treatment management. However, there is still disagreement in the field on the benefits of the procedure. Three different meta-analyses carried out on preoperative embolization in carotid body tumor surgery have reported different and controversial results^5–7^. According to two of the meta-analyses, surgical resection of the tumor after preoperative embolization appeared to shorten the duration of surgery and reduce blood loss compared with surgery without preoperative embolization^5,7^. However, the third meta-analysis reported that preoperative embolization did not provide sufficient benefit^6^. Cobb et al. have also reported that embolization was not beneficial^8^. Upon the publication of these studies, some institutions carried out surgical resections without preoperative embolization; however, there was significant blood loss in these patients^9^.

Several studies have examined the efficacy of preoperative embolization. Some studies have demonstrated that preoperative embolization decreases blood loss during surgery and provides easy dissection of the tumor from the internal/external carotid arterial wall^10^. However, other studies have reported that preoperative embolization is ineffective^11–13^. Therefore, it is imperative to understand the reasons for the differences in the effectiveness of preoperative embolization in the different studies. Shiga et al. reported that the timing of pre-embolization may affect surgical outcomes^14^. Katagiri et al. reported that same-day preoperative embolization significantly decreased blood loss and surgery time^15^.

Another reason for the different outcomes observed in the different studies could be the success rate of embolization and the experience of the interventional radiologist. The percentage of devascularization after embolization may also affect surgical outcomes. However, to the best of our knowledge, none of the studies in the published literature have evaluated this. Therefore, the purpose of this study was to determine the effect of the percentage of tumor devascularization achieved by preoperative embolization on surgical outcomes.

## METHODS

### Study population

Ethical approval for this retrospective and cross-sectional study was obtained from the local ethics committee (approval number: 2024/03-54). A total of 31 consecutive patients who were diagnosed with histopathological carotid body tumors between 2013 and 2024 were identified from the hospital database. All patients underwent preop embolization and subsequent surgical resection. Notably, 31 patients with 31 carotid body paragangliomas were included in the study.

### Shamblin classification

The Shamblin classification of carotid body tumors in all patients was carried out using preoperative contrast-enhanced neck computed tomography (CT) images. The classification was carried out according to the circumferential contact angle of the tumor with the ICA with group I: <180°, group II: <180–270°, and group III: >270° encasement^4^.

### Preoperative embolization

**Figure 1:**
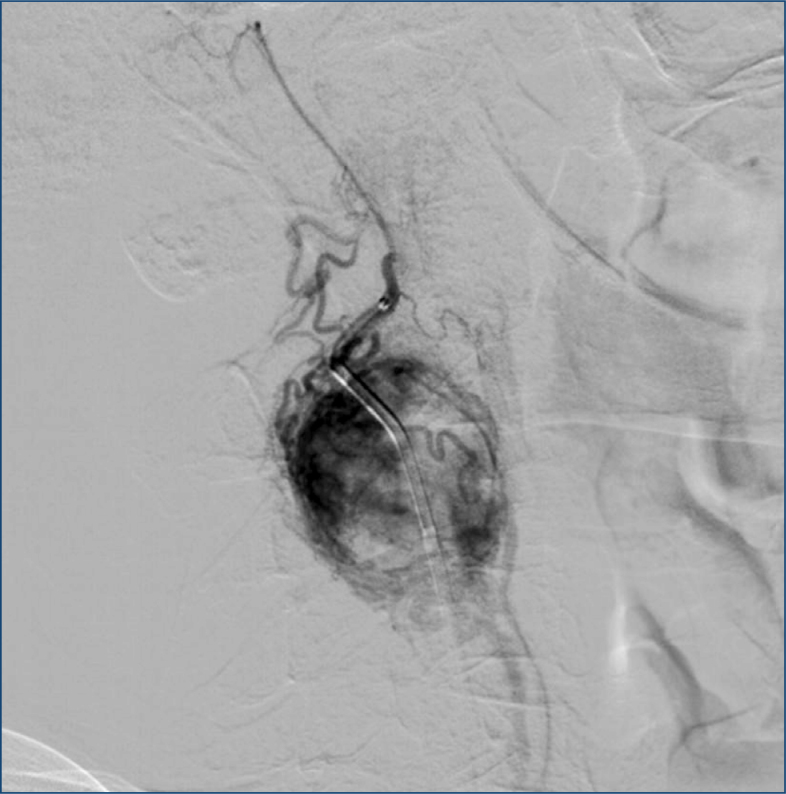
Vascularization of the carotid body tumor before embolization procedure.

**Figure 2:**
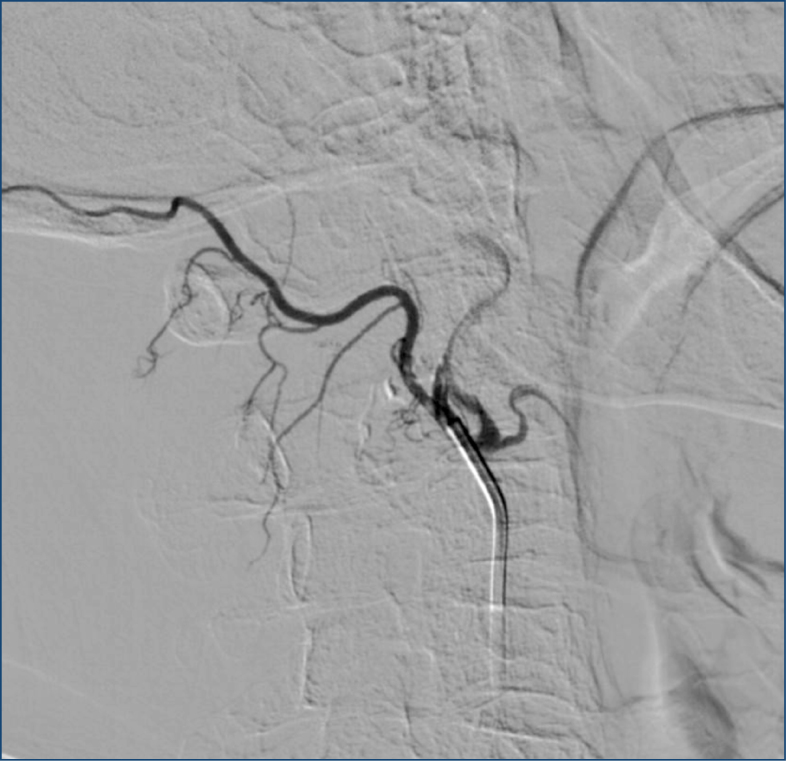
Near-complete devascularization achieved after the embolization procedure in the same patient.

For preoperative embolization, the right femoral artery was punctured, and a 5-Fr sheath was inserted. A 5-Fr diagnostic catheter was next inserted into the common carotid artery (CCA), followed by selective angiography of the ECA and CCA. A microcatheter and a 0.018-inch guide wire were used to carry out super-selective catheterization of the arteries supplying the tumor. Polyvinyl alcohol (PVA) was used as an embolizing agent. PVA particles were mixed with a contrast agent in a 1:1 ratio and injected via the microcatheter. A final angiogram was carried out to assess the degree of embolization and patency of the ICA. The percentage of devascularization was determined by comparing the angiograms before and after embolization by a vascular interventional radiologist (HY). A devascularization rate greater than 50% was accepted as technical success. A devascularization rate of >90% was considered a near-complete embolization (Figures 1 and 2). A devascularization rate of <90% was considered incomplete embolization.

### Surgical resection

Surgery was planned within 24 h after the embolization. The surgical procedure was performed by an ear, nose, and throat specialist under general anesthesia.

Intraoperative and postoperative complications were determined from the hospital database. Hemoglobin loss in blood tests performed immediately before and after the surgery provided information about the amount of blood lost during the surgery.

### Tumor volume

The volume of the paraganglioma was calculated using neck CT images preoperatively. The volume of the carotid body tumor was measured using longitudinal (a), transverse (b), and thick diameter (c). The postoperative volume of the paraganglioma was predicted using surgically resected specimens. Preoperative and postoperative tumor volumes were calculated with the formula: a × b × c × 0.523. Following this, the percentage reduction in tumor volume after surgery was calculated.

### Statistical analysis

After the embolization procedure, patients were divided into two groups: patients with near-complete embolization (devascularization rate >90%) and patients with incomplete embolization (devascularization rate <90%). The demographic and surgical outcomes of the two groups were compared statistically using IBM SPSS, version 25.0. The Kolmogorov-Smirnov test was applied to evaluate whether the distribution was normal. A student t-test was used for normally distributed parameters. A chi-square test was used to compare complication rates.

## RESULTS

A total of 31 patients were included in this retrospective study. With preoperative embolization, 29 patients had a devascularization rate of more than 50%. The technical success rate of preoperative embolization was 93.54%. The devascularization rate achieved in 21 patients (67.7%) was greater than 90%, and these patients were included in the near-complete embolization group. The devascularization rate was less than 90% in 10 patients (32.2%), and these patients were included in the incomplete embolization group. No significant difference was observed between the two groups in terms of age, gender, tumor location (right vs. left), Shamblin classification, and preoperative tumor volume (p>0.05) (Table 1).

**Table 1:** The comparison of demographic data and tumor features of the two groups.

		Near-complete embolization (n=21)	Incomplete embolization (n=10)	p
Age		53.73±14.43	56.80±14.75	0.58
Gender (M/F)		6/15	3/7	1.000
Tumor location (right/left)		13/8	7/3	1.000
Preoperative tumor volume (mL)		26.80±24.97	29.50±16.99	0.761
Shamblin classification				
	Group I (n)	6	3	0.995
	Group II (n)	13	6	
	Group III (n)	2	1	

In the near-complete embolization group, the average volume loss of tumors after surgery was 51.45%. In the group with incomplete devascularization, the average volume loss of the tumor was 50.55%. Regarding tumor volume loss, there was no significant difference between the two groups (p=0.488). However, tumor volume loss was greater in the near-complete embolization group than in the incomplete embolization group.

The mean hemoglobin loss of all patients after surgery was 1.50±1.13. The mean hemoglobin loss after surgery was 1.32±1.13 in patients with near-complete embolization and 1.71±1.16 in patients with incomplete embolization. No significant difference was observed in hemoglobin loss between the two groups. However, hemoglobin loss in the near-complete embolization group was less than in the incomplete embolization group.

Major vascular complications were detected in a total of three patients (9.67%). No major vascular complications were detected in patients with near-complete embolization. In the group with incomplete devascularization, two patients had intraoperative carotid artery injury, and one patient had postoperative hematoma. The complication rate was significantly lower in the group with near-complete embolization (p=0.027). The carotid artery injury in two patients was repaired by a cardiovascular surgeon, while the patient with a postoperative hematoma underwent surgical drainage.

Postoperative neurological complications developed in seven patients (22.58%). Vagus paralysis occurred in four patients, and hypoglossal paralysis occurred in three patients. Temporary nerve paralysis occurred in five patients, and permanent nerve paralysis occurred in two patients. Neurological complications occurred in five patients (23.80%) in the near-complete embolization group and in two patients (20%) in the incomplete embolization group. No significant difference was observed in terms of neurological complications between the two groups (p=0.813).

## DISCUSSION

Carotid body tumors require surgical resection due to the possibility of growth and local invasion. Because of the high-grade vascularization of the tumor, surgical resection carries a considerable risk of blood loss. Thus, preoperative embolization is a useful approach to reduce the risk of bleeding^10^. In this study, all patients underwent preoperative embolization. No vascular complications were observed in the group with near-complete embolization (devascularization rate >90%). The vascular complication rate was 30% in the incomplete embolization group (devascularization rate <90%). In the near-complete embolization group, the vascular complication rate was significantly lower. According to our study, the preoperative devascularization rate should be at least 90% to minimize the risk of vascular complications.

Presurgical embolization for highly vascular tumors has been used for the past 30 years^16^. Preoperative embolization of paragangliomas is widely implemented as it reduces intraoperative blood loss, decreases tumor volume, increases intraoperative tumor visualization, and facilitates tumor dissection^17^. In our study, vascular complications developed in three patients with incomplete embolization. Of these, two patients had intraoperative carotid artery injuries, while postoperative hematoma was detected in one patient. Our data suggest that nearly complete embolization can improve visualization of the tumor and reduce the risk of carotid artery injury.

Patients with preoperative embolization were shown to have a shorter duration of surgery and less blood loss compared with patients without embolization^5^. However, another study reported no intraoperative or postoperative advantage to patients undergoing preoperative embolization^6^. The reason for this difference may be related to the devascularization rates achieved with embolization. We observed significantly lower rates of vascular complications when almost complete devascularization was achieved. However, there was no significant difference in tumor volume decrease, hemoglobin loss, or postoperative neurological complications between the near-complete embolization group and the incomplete embolization group. In this study, the mean hemoglobin loss after surgery was 1.50±1.13. None of the patients required a blood transfusion after surgery.

Embolization with PVA particles provides capillary occlusion to achieve complete or near-complete embolization in cases with prominent arterial feeders. However, sometimes the embolization procedure can be incomplete and time-consuming. The reasons for this are the multiplicity, tortuosity, and small caliber of the feeding arteries. Additional factors include vascular spasticity caused by catheter manipulation and blood supply from the ICA and vertebral artery^18^.

We observed that complete or nearly complete embolization was necessary to minimize the risk of vascular complications. However, total devascularization cannot be achieved with an angiographic embolization procedure in every case. We achieved near-total devascularization in 21 patients (67.7%) and incomplete devascularization in 10 patients (32.2%). In addition, the transarterial embolization procedure has a potential risk of stroke due to the migration of the embolizing agent into the intracranial circulation via collaterals^19^. Due to these disadvantages of the intravascular approach, embolization of paragangliomas with direct puncture is being used at some institutions.

Ozyer et al. used an ultrasound-guided intratumoral injection of n-butyl cyanoacrylate to achieve complete devascularization in patients with incomplete devascularization achieved by transarterial embolization^20^. Pérez-García et al. carried out preoperative embolization of carotid body tumors in six patients by direct puncture using the Squid^®^ embolizing agent. These authors reported near-complete embolization in all cases^21^. A meta-analysis by Schartz et al. showed that the rate of total devascularization was higher in the direct percutaneous puncture approach compared with transarterial embolization^22^. Therefore, the direct puncture approach may be more effective than transarterial embolization in reducing vascular complications. Large-scale and prospective studies are needed to evaluate this.

The limitations of this study include the small number of patients and its retrospective design.

Overall, this study showed that preoperative embolization of carotid body tumors can be effective in preventing complications. Preoperative total or near-total devascularization should be achieved to minimize the risk of vascular complications.
